# Supplementary material for: Reimagining the role of teaching-focused faculty in research-intensive universities: The evolution of scholarly expectations and departmental influence
Source: PLoS One. 2025 Oct 16;20(10):e0334895. doi: 10.1371/journal.pone.0334895 (PMC12530556; doi:10.1371/journal.pone.0334895)
Supplement: S1 Table — (DOCX) [file pone.0334895.s001.docx]

**S1 Table. Survey Questions and Response Types**

| **Demographic Information** | | |
| --- | --- | --- |
| Demographic information. Participants were asked to provide basic demographic information including gender identity, ethnicity/race, college-graduate status, UC campus, department, rank, and previous training. | | |
| **Question** |  | **Response Type** |
| Which of the following best describes your current gender identity? |  | Multiple choice |
| Please specify your ethnicity/race. |  | Select all that apply |
| Are you part of the first generation in your family to graduate college? By this we mean the first in your family to complete a 4-year degree program. |  | Multiple choice |
| At which University of California Campus are you employed? |  | Open-ended response |
| What is your discipline (Home Department)? |  | Open-ended response |
| What best describes your current L(P)SOE title? Please note we use the term PoT throughout this survey to refer to these faculty of all ranks (Assistant PoT, Associate PoT, Full PoT) |  | Multiple choice |
| Which of these formal trainings do you have in your discipline (i.e. the discipline in your home department)? |  | Select all that apply |
| In what year were you hired as a PoT? |  | Open-ended response |
| **Job Expectations** | | |
| Participants were asked to provide an approximation of the percentage of time they believed they spent participating in the three domains of responsibility: teaching, scholarship, and service. Furthermore, we acquired information on the types of scholarly activities (e.g., generating peer reviewed publications, developing undergraduate curriculum, increasing departmental grant funds, etc.) PoTs perceived they accomplished during their time in the PoT position. | | |
| **Question** |  | **Response Type** |
| How much time (%) are PoTs in your department expected to spend engaged in each of the following. Your answers must add up to 100%. - Scholarly work/professional activities |  | Sliding scale 0-100 |
| How much time (%) are PoTs in your department expected to spend engaged in each of the following. Your answers must add up to 100%. - Service |  | Sliding scale 0-100 |
| How much time (%) are PoTs in your department expected to spend engaged in each of the following. Your answers must add up to 100%. - Teaching |  | Sliding scale 0-100 |
| How much time (%) do you spend engaged in each of the following. Your answers must add up to 100%. - Scholarly work/professional activities |  | Sliding scale 0-100 |
| How much time (%) do you spend engaged in each of the following. Your answers must add up to 100%. - Service |  | Sliding scale 0-100 |
| How much time (%) do you spend engaged in each of the following. Your answers must add up to 100%. - Teaching |  | Sliding scale 0-100 |
| Which of the following activities do you perform to fulfill your scholarship/professional responsibilities? |  | Select all that apply |
| How has the reality of being a PoT been different from your expectations from when you were hired? |  | Open-ended response |
| **Resources Available** | | |
| Participants were asked to report on available resources such as training and support around scholarly activities, material support, start-up funds, etc. | | |
| **Question** |  | **Response Type** |
| In your department, which of the following resources or sources of support are available for you to pursue scholarly work? |  | Select all that apply & Open-ended response |
| In your department, which of the following resources or sources of support are available for you to pursue teaching work? |  | Select all that apply |
| My campus provides adequate training opportunities for me to improve my teaching |  | Likert-scale |
| My campus provides adequate training opportunities for me to improve my skills related to my scholarly work |  | Likert-scale & Open-response |
| The amount of my start-up fund (in thousands of dollars), not including housing allowance was: |  | Open-ended response |
| Were you offered research or “lab” space (any additional personal space for group members to work outside of your faculty office) by your department? |  | Multiple choice |
| **Professional Identity** | | |
| Participants were asked about the degree to which they identified as an instructor and separately as a researcher. | | |
| **Question** |  | **Response Type** |
| How much do you identify as a researcher? |  | Likert-scale |
| How much do you identify as an instructor? |  | Likert-scale |
| **Influence on Colleagues' Teaching** | | |
| To determine to what degree PoTs believed they influenced their colleagues. To address this, we developed three items to measure influence: “How much are you influencing your colleagues’ teaching beliefs?”, “How much are you influencing your colleagues’ teaching knowledge,” and “How much are you influencing your colleagues’ teaching practices”. Each item was rated on a scale of 1 (not at all) to 6 (completely). | | |
| **Question** |  | **Response Type** |
| How much are you influencing your colleagues’ teaching beliefs? |  | Likert-scale |
| How much are you influencing your colleagues’ teaching knowledge? |  | Likert-scale |
| How much are you influencing your colleagues’ teaching practices? |  | Likert-scale |
| **Training** | | |
| To determine what forms of training PoTs have received prior to taking on this role, we asked the following questions: “Which of these formal trainings do you have in your discipline (i.e. the discipline in your home department)?” and “Which degree of formal training in education do you have?”. Each of these listed degrees from Master’s – Postdoctoral work | | |
| **Question** |  | **Response Type** |
| Which of these formal trainings do you have in your discipline (i.e. the discipline in your home department)? |  | Select all that apply |
| Which degree of formal training in education do you have? |  | Select all that apply |
