# Supplementary material for: Reimagining the role of teaching-focused faculty in research-intensive universities: The evolution of scholarly expectations and departmental influence
Source: PLoS One. 2025 Oct 16;20(10):e0334895. doi: 10.1371/journal.pone.0334895 (PMC12530556; doi:10.1371/journal.pone.0334895)
Supplement: S2 Table — (DOCX) [file pone.0334895.s002.docx]

**S2 Table. Scholarly activities as predictors of an individuals’ research identity.**

| **Variable** | **β** | **S.E.** | **t-value** | **P-value** |  |
| --- | --- | --- | --- | --- | --- |
| Intercept | 2.49 | 1.00 | 2.49 | 0.01 | * |
| **Activities** |  |  |  |  |  |
| Discipline-specific research | 0.21 | 0.30 | 0.70 | 0.49 |  |
| Discipline-based education research or education research | 0.88 | 0.32 | 2.75 | 0.01 | ** |
| Mentoring undergraduate/graduate student research | 0.38 | 0.32 | 1.19 | 0.24 |  |
| Generating peer-reviewed publications | -0.12 | 0.33 | -0.36 | 0.73 |  |
| Improving teaching practices in the department | -0.55 | 0.40 | -1.38 | 0.17 |  |
| Assessment of teaching/education in the department/campus | -0.06 | 0.30 | -0.20 | 0.84 |  |
| Providing professional development for graduate students | 0.48 | 0.35 | 1.37 | 0.17 |  |
| Providing professional development for K–12 teachers | -0.14 | 0.43 | -0.33 | 0.74 |  |
| Developing undergraduate curriculum | 0.06 | 037 | 0.16 | 0.88 |  |
| **Gender** |  |  |  |  |  |
| Cis-gender female/woman | 0.39 | 0.33 | 1.18 | 0.24 |  |
| Genderqueer, gender non-binary, or gender fluid | 0.57 | 1.26 | 0.45 | 0.65 |  |
| **Ethnicity** |  |  |  |  |  |
| Black or African American | -1.68 | 1.76 | -0.95 | 0.34 |  |
| Asian | 0.23 | 0.66 | 0.35 | 0.72 |  |
| Hispanic or Latina/o/x | 0.07 | 0.64 | 0.11 | 0.91 |  |
| Multiethnic | -0.11 | 0.54 | -0.20 | 0.84 |  |
| Other | 1.70 | 1.22 | 1.39 | 0.17 |  |
| **Campus** |  |  |  |  |  |
| University 2 | 0.42 | 0.84 | 0.50 | 0.62 |  |
| University 3 | -0.17 | 0.82 | -0.21 | 0.84 |  |
| University 4 | 0.50 | 0.90 | 0.56 | 0.58 |  |
| University 5 | 1.37 | 0.93 | 1.47 | 0.14 |  |
| University 6 | 0.76 | 0.79 | 0.96 | 0.34 |  |
| University 7 | 0.91 | 0.81 | 1.12 | 0.25 |  |
| University 8 | 0.94 | 0.93 | 1.01 | 0.32 |  |
| University 9 | 0.19 | 0.99 | 0.19 | 0.85 |  |
| **Department** |  |  |  |  |  |
| Physical Sciences | -0.60 | 0.73 | -0.82 | 0.17 |  |
| Social Sciences | -0.14 | 0.46 | -0.30 | 0.77 |  |
| Computer Science/Engineering | -0.16 | 0.39 | -0.41 | 0.68 |  |
| Other STEM | 0.42 | 0.61 | 0.69 | 0.49 |  |
| **Faculty Rank** |  |  |  |  |  |
| Rank | -0.28 | 0.30 | -0.93 | 0.34 |  |
| A Multiple linear regression analysis was run to assess scholarly activities as predictors of an individual’s research identity (*p<0.05, **p<0.01, ***p<0.001). Residual standard error: 1.596 on 122 degrees of freedom (26 observations deleted due to missingness). Multiple R-squared: 0.2433. Adjusted R-squared: 0.05727. F-statistic: 1.308 on 30 and 122 DF. p-value: 0.1562. | | | | | |
