# Supplementary material for: Reimagining the role of teaching-focused faculty in research-intensive universities: The evolution of scholarly expectations and departmental influence
Source: PLoS One. 2025 Oct 16;20(10):e0334895. doi: 10.1371/journal.pone.0334895 (PMC12530556; doi:10.1371/journal.pone.0334895)
Supplement: S3 Table — Reduced regression model including only scholarly activity predictors of research identity. This model is presented for comparison with the full model (S2 Table). Demographic and contextual controls were excluded in this model. (DOCX) [file pone.0334895.s003.docx]

**S3 Table: Reduced Regression model for Research Identity.**

| **Variable** | **β** | **S.E.** | **t-value** | **P-value** |  |
| --- | --- | --- | --- | --- | --- |
| Intercept | 2.59 | 0.43 | 5.99 | 1.63e-08 | *** |
| **Activities** |  |  |  |  |  |
| Discipline-specific research | 0.13 | 0.27 | 0.48 | 0.63 |  |
| Discipline-based education research or education research | 1.02 | 0.30 | 3.46 | 7.01e-03 | *** |
| Mentoring undergraduate/graduate student research | 0.29 | 0.29 | 0.99 | 0.32 |  |
| Generating peer-reviewed publications | 0.12 | 0.31 | 0.39 | 0.70 |  |
| Improving teaching practices in the department | -0.39 | 036 | -1.08 | 0.28 |  |
| Assessment of teaching/education in the department/campus | 0.12 | 0.29 | 0.42 | 0.68 |  |
| Providing professional development for graduate students | 0.43 | 0.31 | 1.39 | 0.17 |  |
| Providing professional development for K–12 teachers | -0.08 | 0.41 | -0.20 | 0.84 |  |
| Developing undergraduate curriculum | 0.07 | 0.34 | 0.21 | 0.83 |  |
| A Multiple linear regression analysis was run to assess scholarly activities as predictors of an individual’s research identity (*p<0.05, **p<0.01, ***p<0.001). Residual standard error: 1.597 on 145 degrees of freedom (4 observations deleted due to missingness). Multiple R-squared: 0.136 Adjusted R-squared: 0.083. F-statistic: 2.544 on 9 and 145 DF. p-value: 0.010. | | | | | |
