# Supplementary material for: Reimagining the role of teaching-focused faculty in research-intensive universities: The evolution of scholarly expectations and departmental influence
Source: PLoS One. 2025 Oct 16;20(10):e0334895. doi: 10.1371/journal.pone.0334895 (PMC12530556; doi:10.1371/journal.pone.0334895)
Supplement: S5 Table — Reduced regression model including only scholarly activity predictors of perceived influence on teaching. This model is presented for comparison with the full model (S3 Table). Demographic and contextual controls were excluded in this model. (DOCX) [file pone.0334895.s005.docx]

**S4 Table. Scholarly activities as predictors of an individual’s perceived influence on colleagues’ teaching.**

| **Variable** | **β** | **S.E.** | **t-value** | **P-value** |  |
| --- | --- | --- | --- | --- | --- |
| Intercept | 2.85 | 0.59 | 4.83 | 4.81e-06 | *** |
| **Activities** |  |  |  |  |  |
| Discipline-specific research | -0.02 | 0.18 | -0.84 | 0.93 |  |
| Discipline-based education research or education research | 0.44 | 0.20 | 2.19 | 0.03 | * |
| Mentoring undergraduate/graduate student research | -0.45 | 0.19 | -2.35 | 0.02 | * |
| Generating peer-reviewed publications | 0.36 | 0.20 | 2.73 | 0.01 | ** |
| Improving teaching practices in the department | 0.03 | 0.24 | 0.13 | 0.89 |  |
| Assessment of teaching/education in the department/campus | -0.07 | 0.19 | -0.35 | 0.73 |  |
| Providing professional development for graduate students | -0.02 | 0.21 | -0.11 | 0.91 |  |
| Providing professional development for K–12 teachers | 0.10 | 0.27 | 0.37 | 0.71 |  |
| Developing undergraduate curriculum | -0.16 | 0.24 | -0.66 | 0.51 |  |
| **Gender** |  |  |  |  |  |
| Cis-gender female/woman | -0.15 | 0.20 | -0.76 | 0.45 |  |
| Genderqueer, gender non-binary, or gender fluid | -0.93 | 0.72 | -1.30 | 0.20 |  |
| **Ethnicity** |  |  |  |  |  |
| Asian | -0.17 | 0.38 | -0.45 | 0.66 |  |
| Hispanic or Latina/o/x | 0.05 | 0.31 | 0.15 | 0.88 |  |
| Multiethnic | -0.07 | 0.70 | -0.10 | 0.92 |  |
| Other | -0.11 | 0.54 | -0.20 | 0.84 |  |
| **Campus** |  |  |  |  |  |
| University 2 | -0.78 | 0.49 | -0.97 | 0.33 |  |
| University 3 | -0.12 | 0.48 | -02.6 | 0.80 |  |
| University 4 | 0.18 | 0.53 | 0.34 | 0.73 |  |
| University 5 | 0.43 | 0.53 | 0.81 | 0.42 |  |
| University 6 | -0.21 | 0.46 | -0.47 | 0.64 |  |
| University 7 | -0.33 | 0.47 | -0.69 | 0.49 |  |
| University 8 | -0.04 | 0.53 | -0.07 | 0.94 |  |
| University 9 | 0.77 | 0.30 | 1.26 | 0.21 |  |
| **Department** |  |  |  |  |  |
| Physical Sciences | -0.14 | 0.25 | -0.54 | 0.59 |  |
| Social Sciences | -0.10 | 0.29 | -0.36 | 0.72 |  |
| Computer Science/Engineering | 0.03 | 0.24 | 0.13 | 0.90 |  |
| Other STEM | -0.24 | 0.37 | -0.65 | 0.51 |  |
| **Faculty Rank** |  |  |  |  |  |
| **Rank** | 0.04 | 0.18 | 0.20 | 0.84 |  |
| A Multiple linear regression analysis was run to assess scholarly activities as predictors of an individual’s perceived teaching influence (*p<0.05, **p<0.01, ***p<0.001). Residual standard error: 0.90 on 104 degrees of freedom (26 observations deleted due to missingness). Multiple R-squared: 0.297. Adjusted R-squared: 0.108. F-statistic: 1.568 on 28 and 104 DF. p-value: 0.053. | | | | | |
