# Supplementary material for: Reimagining the role of teaching-focused faculty in research-intensive universities: The evolution of scholarly expectations and departmental influence
Source: PLoS One. 2025 Oct 16;20(10):e0334895. doi: 10.1371/journal.pone.0334895 (PMC12530556; doi:10.1371/journal.pone.0334895)
Supplement: Attachment — Submitted filename: Teaching professors.docx [file pone.0334895.s007.docx]

Citations are incorrect for Plos One

Each paragraph should be indent 5 spaces

Please check authors information for the journal

Your introduction is one l. o. n. g paragraph—please break it up into more coherent smaller paragraphs

Line 77 is influenced by

I often use Brofenbrenner in my own research, but I think you need to expand on his systems and how they impact teaching and learning in the university

Need a procedure section

How recruited

What platform was the survey on

Characteristics of the survey

More info on survey development

Table 2 should include the t test information and p values

You need to report checking the assumptions of your multiple regression

Also how did you enter variables? What method did you use?

Adjusted R squares are always equal to or less than the R squared

Would be helpful also to have the t values for each predictor variable as well as the overall ANOVA for the model

From the note at the bottom it seems that your model is not significant

Seems you need to eliminate predictors and do some model building rather than entering all variables at the same time (I am assuming you did that)

Results sections do not have citations—only results

Discussion

“Many of the current STEM educational approaches result in inequitable retention outcomes for 396 students of different demographic backgrounds. “

I don’t see evidence of this in your data and there is no citation – so what do you mean here

The first paragraph in the discussion seems to general and not helpful in understanding how your data connects to your theoretical framework

The drop in discipline research seems more related to not having time to do their reearach---it seems that the Assistants were trained as any research tenure track faculty—but got more teaching responsibilities

In many universities more senior faculty do not publish as much as the newer faculty—but you did not make that comparison, so you don’t know if this is the same issue

It seems to be that these Assistant teaching faculty function like many teaching faculty at non-R-1 institutions

Lines 440 to 446 would be a good place to bring back in your Theoretical framework

Recommendations is one long paragraph---please make paragraphs relating to topic sentences
