# Supplementary material for: Reimagining the role of teaching-focused faculty in research-intensive universities: The evolution of scholarly expectations and departmental influence
Source: PLoS One. 2025 Oct 16;20(10):e0334895. doi: 10.1371/journal.pone.0334895 (PMC12530556; doi:10.1371/journal.pone.0334895)
Supplement: Attachment — Submitted filename: Response to Reviewers.doc [file pone.0334895.s009.doc]

July 03, 2025

PLOS

1265 Battery Street, Suite 200

San Francisco, CA 94111

Dear Dr. Clark and Reviewers,

Thank you for your thoughtful and constructive feedback on our manuscript, *"Reimagining the role of teaching-focused faculty in research-intensive universities: The evolution of scholarly expectations and departmental influence”.* We appreciate the opportunity to revise and resubmit our work to PLOS ONE. We have carefully considered each comment and have made substantial revisions to strengthen the manuscript's clarity, methodological transparency, and overall contribution.

This response letter is organized to address all editorial and reviewer comments in a point-by-point format. For each comment, we have provided a description of the changes made, as well as a rationale for our approach when clarification or respectful disagreement was warranted. Major changes include the addition of a Procedures section, clarification of our mixed methods design, expanded statistical reporting including assumption checks and reduced models, and alignment with PLOS ONE formatting and citation guidelines.

In the following pages, you will find detailed responses to your comments and suggestions. We believe these revisions significantly improve the quality and clarity of the manuscript, and we are grateful for the guidance that led to these improvements.

Sincerely,

Alex Paine, Ph.D.	      	Brian Sato, Ph.D.

Department of Biology, Baylor University	Division of Teaching Excellence and Innovation, University of California, Irvine

Email: [alex_paine@baylor.edu](mailto:alex_paine@baylor.edu)	Email: [bsato@uci.edu](mailto:bsato@uci.edu)

PONE-D-25-02548

Reimagining the Role of Teaching-Focused Faculty in Research-Intensive Universities: The Evolution of Scholarly Expectations and Departmental Influence

PLOS ONE

**Dear Dr. Paine,**

Thank you for submitting your manuscript to PLOS ONE. After careful consideration, we feel that it has merit but does not fully meet PLOS ONE’s publication criteria as it currently stands. Therefore, we invite you to submit a revised version of the manuscript that addresses the points raised during the review process.

Thanks for submitting you article to PLOS ONE. It has been reviewed by two individuals, one who has a review similar to mine and one accept. You need to check the Information for Authors for citations, formatting etc as your manuscript does not follow the guidelines

In addition, it is difficult to understand your analyses as presented.

- Thank you for your feedback. We have responded to initial points regarding analyses below.

Finally, in the discussion and limitations from the findings it seems to me that these teaching professors are not really teaching professors. --they just needed a job. They are more similar to research professors without the support. They are more like those who select to go to traditional teaching colleges. Please discuss the possibility of this in your discussion.

- We somewhat disagree with this point - while true, the PoTs in our sample do have similar training to research faculty, this does not necessarily mean that they did not want their role and/or just needed a job. This is a factor of both what search committees were looking for in candidates (often individuals with similar training as research faculty - as we have seen in prior work by Harlow et. al. 2022) as well as the fact that there currently is not a separate mechanism to train teaching-focused faculty who must possess both disciplinary expertise as well as education-expertise.
- We do agree that if the goal is to bring in more education-trained individuals within this role stakeholders must adjust their hiring expectations. We bring up this point in the *Discussion* (*Recommendations for Hiring*).

Please submit your revised manuscript by Jun 05 2025 11:59PM. If you will need more time than this to complete your revisions, please reply to this message or contact the journal office at plosone@plos.org. When you're ready to submit your revision, log on to [https://www.editorialmanager.com/pone/](https://nam02.safelinks.protection.outlook.com/?url=https%3A%2F%2Furldefense.com%2Fv3%2F__https%3A%2Fwww.editorialmanager.com%2Fpone%2F__%3B!!CzAuKJ42GuquVTTmVmPViYEvSg!K_gEeZ_QPmKwU9Y4pPYFgLrfscfOKRf3aYyU9pnljmZ3BxuCPcY3ANbxHALjAWq81uzj_QSjzqHfCC6j0Jp6$&data=05|02|Alex_Paine@baylor.edu|27481fc8fa8e424a5b8408dd8f4f3796|22d2fb35256a459bbcf4dc23d42dc0a4|0|0|638824292897658620|Unknown|TWFpbGZsb3d8eyJFbXB0eU1hcGkiOnRydWUsIlYiOiIwLjAuMDAwMCIsIlAiOiJXaW4zMiIsIkFOIjoiTWFpbCIsIldUIjoyfQ%3D%3D|0|||&sdata=N%2BR3wBnpn9raQhZE%2BrNSAZSwkPwY%2FPXnnMPQjJzeLfw%3D&reserved=0) and select the 'Submissions Needing Revision' folder to locate your manuscript file.

 Please include the following items when submitting your revised manuscript:

- A rebuttal letter that responds to each point raised by the academic editor and reviewer(s). You should upload this letter as a separate file labeled 'Response to Reviewers'.
- A marked-up copy of your manuscript that highlights changes made to the original version. You should upload this as a separate file labeled 'Revised Manuscript with Track Changes'.
- An unmarked version of your revised paper without tracked changes. You should upload this as a separate file labeled 'Manuscript'.

If you would like to make changes to your financial disclosure, please include your updated statement in your cover letter. Guidelines for resubmitting your figure files are available below the reviewer comments at the end of this letter.

If applicable, we recommend that you deposit your laboratory protocols in protocols.io to enhance the reproducibility of your results. Protocols.io assigns your protocol its own identifier (DOI) so that it can be cited independently in the future. For instructions see: [https://journals.plos.org/plosone/s/submission-guidelines#loc-laboratory-protocols](https://nam02.safelinks.protection.outlook.com/?url=https%3A%2F%2Furldefense.com%2Fv3%2F__https%3A%2Ftrack.editorialmanager.com%2FCL0%2Fhttps%3A*2F*2Fjournals.plos.org*2Fplosone*2Fs*2Fsubmission-guidelines*23loc-laboratory-protocols%2F1%2F010f01965736a509-fe9d9968-2bc2-4f25-a566-c7ba5d9da885-000000%2FOPeUyivpRIScQp_jGGaLyETCMU-hAjAaRY9lIX7PBLc%3D207__%3BJSUlJSUl!!CzAuKJ42GuquVTTmVmPViYEvSg!K_gEeZ_QPmKwU9Y4pPYFgLrfscfOKRf3aYyU9pnljmZ3BxuCPcY3ANbxHALjAWq81uzj_QSjzqHfCCqOPkr7$&data=05|02|Alex_Paine@baylor.edu|27481fc8fa8e424a5b8408dd8f4f3796|22d2fb35256a459bbcf4dc23d42dc0a4|0|0|638824292897686500|Unknown|TWFpbGZsb3d8eyJFbXB0eU1hcGkiOnRydWUsIlYiOiIwLjAuMDAwMCIsIlAiOiJXaW4zMiIsIkFOIjoiTWFpbCIsIldUIjoyfQ%3D%3D|0|||&sdata=LtgziQAgldCl0Rqj7OvrgbbDFaty4aiqhv9a3j2MjGI%3D&reserved=0). Additionally, PLOS ONE offers an option for publishing peer-reviewed Lab Protocol articles, which describe protocols hosted on protocols.io. Read more information on sharing protocols at [https://plos.org/protocols?utm_medium=editorial-email&utm_source=authorletters&utm_campaign=protocols](https://nam02.safelinks.protection.outlook.com/?url=https%3A%2F%2Furldefense.com%2Fv3%2F__https%3A%2Ftrack.editorialmanager.com%2FCL0%2Fhttps%3A*2F*2Fplos.org*2Fprotocols*3Futm_medium%3Deditorial-email*26utm_source%3Dauthorletters*26utm_campaign%3Dprotocols%2F1%2F010f01965736a509-fe9d9968-2bc2-4f25-a566-c7ba5d9da885-000000%2F_iwfZbftwICKeBEH2wRDomt0lhr31Gkm2PoEy4AfgWw%3D207__%3BJSUlJSUl!!CzAuKJ42GuquVTTmVmPViYEvSg!K_gEeZ_QPmKwU9Y4pPYFgLrfscfOKRf3aYyU9pnljmZ3BxuCPcY3ANbxHALjAWq81uzj_QSjzqHfCOrETNzj$&data=05|02|Alex_Paine@baylor.edu|27481fc8fa8e424a5b8408dd8f4f3796|22d2fb35256a459bbcf4dc23d42dc0a4|0|0|638824292897703574|Unknown|TWFpbGZsb3d8eyJFbXB0eU1hcGkiOnRydWUsIlYiOiIwLjAuMDAwMCIsIlAiOiJXaW4zMiIsIkFOIjoiTWFpbCIsIldUIjoyfQ%3D%3D|0|||&sdata=Np9NpXOuICnnggm0HK8ZjijT7FkDBoGaWhKVkz7r4NI%3D&reserved=0).

We look forward to receiving your revised manuscript.

Kind regards,

Mary Diane Clark, PhD
Academic Editor
PLOS ONE

**Journal Requirements:**

1. When submitting your revision, we need you to address these additional requirements.

Please ensure that your manuscript meets PLOS ONE's style requirements, including those for file naming. The PLOS ONE style templates can be found at

https://journals.plos.org/plosone/s/file?id=wjVg/PLOSOne_formatting_sample_main_body.pdf and

https://journals.plos.org/plosone/s/file?id=ba62/PLOSOne_formatting_sample_title_authors_affiliations.pdf

2. Thank you for stating in your Funding Statement:

This work was supported by the National Science Foundation Grant DUE #1821724.

Please provide an amended statement that declares *all* the funding or sources of support (whether external or internal to your organization) received during this study, as detailed online in our guide for authors at http://journals.plos.org/plosone/s/submit-now. Please also include the statement “There was no additional external funding received for this study.” in your updated Funding Statement.

Please include your amended Funding Statement within your cover letter. We will change the online submission form on your behalf.

- Per the reviewers request, the funding statement ”This work was supported by the National Science Foundation Grant DUE #1821724, There was no additional external funding received for this study” has been removed from the acknowledgements section and is now only found within the cover letter

3. Thank you for stating the following in the Acknowledgments Section of your manuscript:

This work was supported by the National Science Foundation Grant DUE #1821724.

We note that you have provided funding information that is not currently declared in your Funding Statement. However, funding information should not appear in the Acknowledgments section or other areas of your manuscript. We will only publish funding information present in the Funding Statement section of the online submission form.

Please remove any funding-related text from the manuscript and let us know how you would like to update your Funding Statement. Currently, your Funding Statement reads as follows:

This work was supported by the National Science Foundation Grant DUE #1821724.

Please include your amended statements within your cover letter; we will change the online submission form on your behalf.

- Per the reviewers request, the funding statement ”This work was supported by the National Science Foundation Grant DUE #1821724, There was no additional external funding received for this study” has been removed from the acknowledgements section and is now only found within the cover letter

4. We note that this data set consists of interview transcripts. Can you please confirm that all participants gave consent for interview transcript to be published?

- All information collected from faculty was done so through the online survey. Quotes were taken from open-response items to this survey. As such consent was provided prior to taking the survey and all survey participants were deidentified prior to data analysis. Information about these open-ended questions can be found throughout the *Methods* and more text has been added within the manuscript to clarify this form of data collection. Additionally, a statement about deidentification of survey participants has been added to the *Methods* section.

If they DID provide consent for these transcripts to be published, please also confirm that the transcripts do not contain any potentially identifying information (or let us know if the participants consented to having their personal details published and made publicly available). We consider the following details to be identifying information:

- Names, nicknames, and initials

- Age more specific than round numbers

- GPS coordinates, physical addresses, IP addresses, email addresses

- Information in small sample sizes (e.g. 40 students from X class in X year at X university)

- Specific dates (e.g. visit dates, interview dates)

- ID numbers

Or, if the participants DID NOT provide consent for these transcripts to be published:

- Provide a de-identified version of the data or excerpts of interview responses

- Provide information regarding how these transcripts can be accessed by researchers who meet the criteria for access to confidential data, including:

a) the grounds for restriction

b) the name of the ethics committee, Institutional Review Board, or third-party organization that is imposing sharing restrictions on the data

c) a non-author, institutional point of contact that is able to field data access queries, in the interest of maintaining long-term data accessibility.

d) Any relevant data set names, URLs, DOIs, etc. that an independent researcher would need in order to request your minimal data set.

For further information on sharing data that contains sensitive participant information, please see: https://journals.plos.org/plosone/s/data-availability#loc-human-research-participant-data-and-other-sensitive-data

If there are ethical, legal, or third-party restrictions upon your dataset, you must provide all of the following details (https://journals.plos.org/plosone/s/data-availability#loc-acceptable-data-access-restrictions):

a) A complete description of the dataset

b) The nature of the restrictions upon the data (ethical, legal, or owned by a third party) and the reasoning behind them

c) The full name of the body imposing the restrictions upon your dataset (ethics committee, institution, data access committee, etc)

d) If the data are owned by a third party, confirmation of whether the authors received any special privileges in accessing the data that other researchers would not have

e) Direct, non-author contact information (preferably email) for the body imposing the restrictions upon the data, to which data access requests can be sent

- This does not apply to our dataset

5. Please include your full ethics statement in the ‘Methods’ section of your manuscript file. In your statement, please include the full name of the IRB or ethics committee who approved or waived your study, as well as whether or not you obtained informed written or verbal consent. If consent was waived for your study, please include this information in your statement as well.

- This does not apply to our dataset

6. Please include captions for your Supporting Information files at the end of your manuscript, and update any in-text citations to match accordingly. Please see our Supporting Information guidelines for more information: <http://journals.plos.org/plosone/s/supporting-information>.

- A heading has been added to the methods to more clearly present this information

**Additional Editor Comments:**

Important topic as many research trained PhDs are accepting jobs as Teaching faculty most with as little educational backgrounds as those in tenure track research positions.

Reviewer 1 and I have provided many suggestions for improving the manuscript. I have some concerns about how the analyses were conducted as what is provided in the paper is somewhat limited to allow me to evaluate how the statistics were conducted. To avoid this you Data Analyses section needs to be much more detailed to allow the reader to understand your analysis

- We have expanded upon the statistics both in the *Methods* and *Data Analysis* sections of this manuscript.

Please read the comments in the attached file.

In addition, there are three sections that I suggest you eliminate. Figure 1 takes a lot of space and could be conveyed easier in text or at most a table. DEI is not the focus on this manuscript, I suggest you take it out. Then Brofenbrenner does not contribute to this manuscript.

- In response to these comments, we have moved Fig 1 to the supplemental materials and references in the Discussion have been removed. In regards to the use of Brofenbrenner, we use this only to cite how his work has influenced the capacity building framework, but can remove this if the editor prefers.

**Reviewers' comments:**

Reviewer's Responses to Questions

Comments to the Author

1. Is the manuscript technically sound, and do the data support the conclusions?

The manuscript must describe a technically sound piece of scientific research with data that supports the conclusions. Experiments must have been conducted rigorously, with appropriate controls, replication, and sample sizes. The conclusions must be drawn appropriately based on the data presented.

Reviewer #1: Partly

Reviewer #2: Yes

2. Has the statistical analysis been performed appropriately and rigorously?

Reviewer #1: I Don't Know

Reviewer #2: Yes

3. Have the authors made all data underlying the findings in their manuscript fully available?

The [PLOS Data policy](http://www.plosone.org/static/policies.action#sharing) requires authors to make all data underlying the findings described in their manuscript fully available without restriction, with rare exception (please refer to the Data Availability Statement in the manuscript PDF file). The data should be provided as part of the manuscript or its supporting information, or deposited to a public repository. For example, in addition to summary statistics, the data points behind means, medians and variance measures should be available. If there are restrictions on publicly sharing data—e.g. participant privacy or use of data from a third party—those must be specified.

Reviewer #1: Yes

Reviewer #2: Yes

4. Is the manuscript presented in an intelligible fashion and written in standard English?

PLOS ONE does not copyedit accepted manuscripts, so the language in submitted articles must be clear, correct, and unambiguous. Any typographical or grammatical errors should be corrected at revision, so please note any specific errors here.

Reviewer #1: Yes

Reviewer #2: Yes

5. Review Comments to the Author

Please use the space provided to explain your answers to the questions above. You may also include additional comments for the author, including concerns about dual publication, research ethics, or publication ethics. (Please upload your review as an attachment if it exceeds 20,000 characters)

Reviewer #1: The manuscript contains minor grammatical errors that require correction for improved clarity and readability.

- The manuscript has been thoroughly read through to address any grammatical errors.

There are inconsistencies in the formatting and usage of in-text citations that should be addressed.

- Headings of major sections changed to 18pt font and sentence case
- Headings of sub-sections changed to 16pt font … just through methods
- Headings of sub-sections within sub-sections changed to 14pt font
- Document changed to single spaced
- Each paragraph is independent by 5 spaces

The abstract does not include mention of the qualitative or statistical methods used in the study, which may limit readers' understanding of the research design.

- The wording within the abstract has been changed to address this issue

Up to the Methods section, the manuscript lacks any discussion of statistical analysis, which makes the later emergence of a mixed methods approach feel abrupt and disjointed. Earlier sections read as though the study was exclusively qualitative.

- We have added additional references to the survey/quantitative nature of the work prior to the methods to help alleviate this concern within the *Abstract* and *Introduction.*

The Discussion section opens with a focus on diversity, equity, and inclusion (DEI), but these themes are not introduced or addressed elsewhere in the manuscript. As a result, this section appears disconnected and underdeveloped.

- This is a great point and we have adjusted the beginning of the *Discussion* to reflect the need to broadly improve STEM education to support the success of all students.

In the Data Analysis section, the investigators do not address potential statistical relationships between participants’ gender identity and their perceptions of their roles as STEM Teaching Faculty members. Given the near-even gender distribution of participants and the male-dominated nature of STEM fields, exploring this relationship may yield valuable insights.

- We examined gender as a potential predictive variable in Tables 5 and 6, but it was not significant in these analyses.

The research questions are not accompanied by explicitly stated hypotheses. In a mixed methods investigation, articulating at least tentative expectations would strengthen the study’s rationale and coherence

- Thank you for this helpful suggestion. We chose not to include explicit hypotheses due to the exploratory nature of our mixed methods design. The qualitative component followed a phenomenological approach, aiming to capture lived experiences without imposing prior expectations. Our quantitative analyses were similarly exploratory, intended to identify patterns rather than test specific predictions. While we had tentative ideas informed by anecdotal evidence, we lacked sufficient empirical grounding to justify formal hypotheses. We agree that including tentative expectations may enhance clarity and will consider this in future work. A more detailed description of this has been added to the *Data Analysis* section of the manuscript.

For RQ3, the discussion of participants’ influence on colleagues’ teaching lacks cohesion and depth. Additionally, the basis for measuring this influence is unclear, as no data were collected from the colleagues themselves, only three Likert-scale questions of the participants' opinions on their influence were presented. This section appears highly speculative, potentially misleading, and would benefit from further investigation, elaboration and clarification

- We acknowledge that this is a limitation of surveying for self-reported influence, but it is commonly done in similar settings. We also know from our own context that other faculty do view PoTs as influential [1, 13, 14, 15, 27, 28, 29, 30]. This has also been listed as a limitation in the work. As such, we gently push back on the suggestion that these findings are highly speculative and potentially misleading.

**Reviewer #2:** This article highlights the increased reliance of some universities, in this case, within the UC system in STEM fields, upon teaching faculty, that these faculty are expected to and are engaging in significant pedagogy research, and that their pedagogically informed methods are perceived to impact teaching improvements and departmental teaching strategies

The article clearly elaborates theoretical framework, methods, and data collection, as well as results demonstrating that teaching faculty are perceived to contribute to internal capacity for educational change in meeting students’ learning and inclusion needs to a greater degree than research focused colleagues. The article also reveals that teaching faculty report significant concerns that include not being provided with adequate training and resources and feeling less included and respected than research-focused faculty within their departments

Results: RQ1 considers whether an increase has occurred in research expectations for teaching faculty. Results indicate assistant teaching professors report greater research expectations and spending more time on research than their tenured counterparts. The reviewer notes that some mention could be made that this finding might reflect motivation for promotion, in addition to perceived increase in demand

RQ2 considers how “scholarly activities correlate with professional identity and self-reported influence of TP/PoTs.” Findings indicate that assistant teaching professors engaging in educational research report greater identification as researchers than their senior teaching counterparts and that educational publications increased influence more than teaching and mentoring practices.

RQ3 considers preparedness in terms of training and resources, shedding light on the fact that although teaching faculty are expected to focus on that activity and related research and service, few have received training other than the pedagogical research they conduct. Teaching faculty also report inadequate developmental resources due to lack of departmental awareness that pedagogical research is a form of research that also warrants institutional support. The article concludes with appropriate recommendations for teaching faculty and their institutions to explore strategies to improve training and resources. The article sheds light on important issues that affect faculty, students, and academic departments and suggests that this area of inquiry warrants further research, for which this article provides a foundation.

- We sincerely thank Reviewer #2 for their thoughtful and comprehensive summary of our work. We appreciate the recognition of our study’s contribution to understanding the evolving role of teaching-focused faculty, the comments on our theoretical framing and methodology, and the significance of our findings. We also value the suggestion to consider the role of promotion motivation in interpreting RQ1 results and will incorporate this point into our *Discussion*.

6. PLOS authors have the option to publish the peer review history of their article ([what does this mean?](https://journals.plos.org/plosone/s/editorial-and-peer-review-process#loc-peer-review-history)). If published, this will include your full peer review and any attached files.


If you choose “no”, your identity will remain anonymous but your review may still be made public.


**Do you want your identity to be public for this peer review?** For information about this choice, including consent withdrawal, please see our [Privacy Policy](https://www.plos.org/privacy-policy).

Reviewer #1: No

Reviewer #2: No

[NOTE: If reviewer comments were submitted as an attachment file, they will be attached to this email and accessible via the submission site. Please log into your account, locate the manuscript record, and check for the action link "View Attachments". If this link does not appear, there are no attachment files.]

While revising your submission, please upload your figure files to the Preflight Analysis and Conversion Engine (PACE) digital diagnostic tool, <https://pacev2.apexcovantage.com/>. PACE helps ensure that figures meet PLOS requirements. To use PACE, you must first register as a user. Registration is free. Then, login and navigate to the UPLOAD tab, where you will find detailed instructions on how to use the tool. If you encounter any issues or have any questions when using PACE, please email PLOS at figures@plos.org. Please note that Supporting Information files do not need this step.

**Responses to attached document.**

Citations are incorrect for Plos One

- Citations have been changed to follow PLOS one guidelines

Each paragraph should be indent 5 spaces

- This had been corrected

Please check authors information for the journal

Your introduction is one l. o. n. g paragraph—please break it up into more coherent smaller paragraphs

- This has been updated, and the paragraph has been broken into 3 more coherent smaller paragraphs.

Line 77 is influenced by

- This has been updated

I often use Brofenbrenner in my own research, but I think you need to expand on his systems and how they impact teaching and learning in the university

- We are relying more heavily on the capacity building framework (which itself is influenced by ecological systems theory, which is why we do not expand on Brofenbrenner’s work. If the editor prefers, we can remove the Brofenbrenner citation and mention of ecological system’s theory.

Need a procedure section

How recruited

What platform was the survey on

Characteristics of the survey

More info on survey development

- A procedures section has been added to the manuscript. Information on recruitment, characteristics, and survey development can be found in the methods under survey development and data collection within this newly labeled Procedures section. This survey was developed previously by Harlow et. al. Additionally, text from the manuscript has been reordered to more clearly describe the details of the survey used for this research.

Table 2 should include the t test information and p values

- We have added a fourth column to this table that lists the specific p values.

You need to report checking the assumptions of your multiple regression

- For all regression models (Tables 5 and 6), we evaluated and met the major assumptions of linear regression, including linearity, homoscedasticity, normality of residuals, multicollinearity, and independence of errors. Despite minor non-normality, all models are considered robust for interpretation.

**Table 5 (Research Identity):**

- Residuals vs. fitted plots showed acceptable linearity and variance. Q–Q plots showed minor tail deviations; Shapiro–Wilk p < 0.05. GVIFs < 2 indicated no multicollinearity, and the Durbin–Watson statistic (1.80, p = 0.10) supported residual independence.

**Table 6 (Teaching Influence):**

- Thank you for encouraging us to examine these models more closely. We initially constructed three separate models to assess whether scholarly activities predicted TP/PoT faculty members’ perceived influence on (1) teaching beliefs, (2) teaching knowledge, and (3) teaching practices of their colleagues. Upon further analysis, we found that a combined model, using an averaged composite outcome across these three dimensions, offered a better overall fit and yielded more stable results than the individual models.
- Given our broader interest in faculty’s overall influence on colleagues’ teaching, we restructured the analysis to use an average composite outcome that captured general teaching influence across the three dimensions. This revised model demonstrated improved fit and yielded results that approached statistical significance. As such, we report findings based on this composite measure to better reflect the holistic nature of perceived teaching influence, rather than focusing on the three components separately.
- All key regression assumptions were reasonably met. Residuals vs. fitted plots showed no major deviations from linearity or homoscedasticity (Breusch–Pagan p = 0.933). The Q–Q plot showed minor deviation in the upper tail; although the Shapiro–Wilk test was significant (p = 0.003), the distribution was approximately normal. Adjusted GVIF values were all below 2, indicating no multicollinearity concerns. The model is robust for interpretation.

Also how did you enter variables? What method did you use?

- We used the standard “enter” method (simultaneous entry of all predictors) to evaluate the unique contribution of each scholarly activity and demographic variable within a full conceptual model. We acknowledge that this approach may limit statistical power due to the number of predictors relative to the sample size and degrees of freedom.
- Independent variables included:
  - Binary (0/1): Scholarly activities (no/yes)
  - Categorical: Gender, ethnicity, campus, department, and faculty rank
- Dependent variables included:
  - Continuous: Research Identity (1–7 scale; Table 5) and Influence on Teaching (1–6 scale; Table 6)

Adjusted R squares are always equal to or less than the R squared

- We confirm that the adjusted R² values are correctly reported and lower than the multiple R², as expected due to the number of predictors included in the models. These values are accurately interpreted and align with best reporting practices.
- **Table 5: Research Identity**

| R² = 0.243 | Adjusted R² = 0.057 | F(30,122) = 1.31 | p = 0.156 |
| --- | --- | --- | --- |

- **Table 6: Teaching Influence**

| R² = 0.297 | Adjusted R² = 0.108 | F(28, 104) = 1.57 | p=0.053 |
| --- | --- | --- | --- |

Would be helpful also to have the t values for each predictor variable as well as the overall ANOVA for the model…next week's problem

- We have added the *t*-values for each predictor to Tables 5 and 6 to provide a more complete statistical summary of the regression models.
- We thank the reviewer for the suggestion about the use of an overall ANOVA. Type III ANOVA tests were conducted for each regression model to assess the unique contribution of individual predictors. Results showed that discipline-based education research (DBER) was a significant predictor across both models. Additional predictors such as mentoring and peer-reviewed publishing were significant in predicting influence on teaching.

From the note at the bottom it seems that your model is not significant

- **Research Identity (Table 5)**: The overall model was not statistically significant (p = 0.156), though individual predictors such as engaging in discipline-based education research (β = 0.88, p = 0.01) were significant and theoretically meaningful.
- I**nfluence on Teaching (Table 6):** The full model explained approximately 30% of the variance in research identity (*R²* = 0.297; *Adj. R²* = 0.108), and approached overall significance (*F*(28, 104) = 1.57, *p* = 0.053).

Seems you need to eliminate predictors and do some model building rather than entering all variables at the same time (I am assuming you did that)

- Thank you for this suggestion. While our primary analyses used a full model to assess the unique contribution of each scholarly activity while controlling demographics, we agree that model parsimony is important. To address this, we conducted additional reduced-model analyses that excluded demographic variables. These models demonstrated improved fit and are now included in the supplemental materials for reference.

Results sections do not have citations—only results

- As requested, all citations mentioned within the results section have been removed.

**Discussion**

“Many of the current STEM educational approaches result in inequitable retention outcomes for 396 students of different demographic backgrounds.

- This was removed based on reviewer feedback on DEI.

I don’t see evidence of this in your data and there is no citation – so what do you mean here

- Based on feedback from the other reviewer we have removed this information.

The first paragraph in the discussion seems to general and not helpful in understanding how your data connects to your theoretical framework

- This is a great point. We have highlighted the framework more prominently in the first paragraph.

The drop in discipline research seems more related to not having time to do their research---it seems that the Assistants were trained as any research tenure track faculty—but got more teaching responsibilities

- Based on our data, we are not seeing a drop in disciplinary research in exchange for more teaching, but a gain in discipline-based education research for assistant PoTs relative to Assoc/Full PoTs. While we agree that higher teaching responsibilities are likely contributing to lower research productivity in general, our finding is the higher engagement in DBER in more recently hired PoTs as opposed to a teaching for research trade-off.

In many universities more senior faculty do not publish as much as the newer faculty—but you did not make that comparison, so you don’t know if this is the same issue

- This is an interesting idea that we aim to explore in future work. From our self-reported data (Table 3), Assistant level PoTs say they are publishing at the same rate as their Assoc/Full PoT colleagues.

It seems to be that these Assistant teaching faculty function like many teaching faculty at non-R-1 institutions

- This may be the case and is something we plan to explore in the future.

Lines 440 to 446 would be a good place to bring back in your Theoretical framework

- We attempted to do this initially, but have added text to make it more obvious

Recommendations is one long paragraph---please make paragraphs relating to topic sentences

- As per request, this has been updated, and the paragraph has been broken into 3 subsequent paragraphs relating to topic sentences.

Additional Comments to the editor and reviewers.

- Due to a recent official change in title of these faculty, the wording throughout this document has been changed from Teaching Professor (TP)/ Professor of Teaching (PoT) to just Professor of Teaching (PoT)
