# Supplementary material for: Reimagining the role of teaching-focused faculty in research-intensive universities: The evolution of scholarly expectations and departmental influence
Source: PLoS One. 2025 Oct 16;20(10):e0334895. doi: 10.1371/journal.pone.0334895 (PMC12530556; doi:10.1371/journal.pone.0334895)
Supplement: Attachment — Submitted filename: Response to Reviewers_9_25.doc [file pone.0334895.s010.doc]

September 29, 2025

PLOS

1265 Battery Street, Suite 200

San Francisco, CA 94111

Dear Dr. Clark and Reviewers,

Thank you for your thoughtful and constructive feedback on our manuscript, *"Reimagining the role of teaching-focused faculty in research-intensive universities: The evolution of scholarly expectations and departmental influence”.* We appreciate the opportunity to revise and resubmit our work to PLOS ONE. We have carefully considered each comment and have made changes to improve clarity, transparency, and overall contribution of the manuscript.

This response letter is organized to address all editorial and reviewer comments in a point-by-point format. For each comment, we have provided a description of the changes made. Changes include shortening long sentences for clarity, correcting minor textual issues, improving statistical reporting with t-values, degrees of freedom, and multiple-comparison corrections, and moving full regression models to the supplemental materials while summarizing only significant results in the main text.

In the following pages, you will find detailed responses to your comments and suggestions. We believe these revisions significantly improve the quality and clarity of the manuscript, and we are grateful for the guidance that led to these improvements.

Sincerely,

Alex Paine, Ph.D.	      	Brian Sato, Ph.D.

Department of Biology, Baylor University	Division of Teaching Excellence and Innovation, University of California, Irvine

Email: [alex_paine@baylor.edu](mailto:alex_paine@baylor.edu)	Email: [bsato@uci.edu](mailto:bsato@uci.edu)

PONE-D-25-02548R1

Reimagining the Role of Teaching-Focused Faculty in Research-Intensive Universities: The Evolution of Scholarly Expectations and Departmental Influence

PLOS ONE

Dear Dr. Paine,

Thank you for submitting your manuscript to PLOS ONE. After careful consideration, we feel that it has merit but does not fully meet PLOS ONE’s publication criteria as it currently stands. Therefore, we invite you to submit a revised version of the manuscript that addresses the points raised during the review process.

Thank you for the major revision, the paper is more direct and clearer. I am not going to send it out for a second review but I do have a few things I need you to change. When those are completed I will move the paper forward rapidly.

Please submit your revised manuscript by Oct 03 2025 11:59PM. If you will need more time than this to complete your revisions, please reply to this message or contact the journal office at [plosone@plos.org](mailto:plosone@plos.org). When you're ready to submit your revision, log on to https://www.editorialmanager.com/pone/ and select the 'Submissions Needing Revision' folder to locate your manuscript file.

Please include the following items when submitting your revised manuscript:

- A rebuttal letter that responds to each point raised by the academic editor and reviewer(s). You should upload this letter as a separate file labeled 'Response to Reviewers'.
- A marked-up copy of your manuscript that highlights changes made to the original version. You should upload this as a separate file labeled 'Revised Manuscript with Track Changes'.
- An unmarked version of your revised paper without tracked changes. You should upload this as a separate file labeled 'Manuscript'.

If you would like to make changes to your financial disclosure, please include your updated statement in your cover letter. Guidelines for resubmitting your figure files are available below the reviewer comments at the end of this letter.

If applicable, we recommend that you deposit your laboratory protocols in protocols.io to enhance the reproducibility of your results. Protocols.io assigns your protocol its own identifier (DOI) so that it can be cited independently in the future. For instructions see: [https://journals.plos.org/plosone/s/submission-guidelines#loc-laboratory-protocols](https://nam02.safelinks.protection.outlook.com/?url=https%3A%2F%2Ftrack.editorialmanager.com%2FCL0%2Fhttps%3A%252F%252Fjournals.plos.org%252Fplosone%252Fs%252Fsubmission-guidelines%2523loc-laboratory-protocols%2F1%2F010f0198c14e5a17-81576c75-4302-4594-b9c1-ac608eda842f-000000%2FMaWznoRdju3-apLg4g2MZvQMQZZmNsW2IXgW4hgCFf8%3D224&data=05|02|alex_paine@baylor.edu|5537a85f84b441c587ca08dddef4fc97|22d2fb35256a459bbcf4dc23d42dc0a4|0|0|638911865991261079|Unknown|TWFpbGZsb3d8eyJFbXB0eU1hcGkiOnRydWUsIlYiOiIwLjAuMDAwMCIsIlAiOiJXaW4zMiIsIkFOIjoiTWFpbCIsIldUIjoyfQ%3D%3D|0|||&sdata=3aTxpYaWXCG8rLQApUXeR8xlutX4Ut8VI4dpkN2DBaU%3D&reserved=0). Additionally, PLOS ONE offers an option for publishing peer-reviewed Lab Protocol articles, which describe protocols hosted on protocols.io. Read more information on sharing protocols at [https://plos.org/protocols?utm_medium=editorial-email&utm_source=authorletters&utm_campaign=protocols](https://nam02.safelinks.protection.outlook.com/?url=https%3A%2F%2Ftrack.editorialmanager.com%2FCL0%2Fhttps%3A%252F%252Fplos.org%252Fprotocols%253Futm_medium%3Deditorial-email%2526utm_source%3Dauthorletters%2526utm_campaign%3Dprotocols%2F1%2F010f0198c14e5a17-81576c75-4302-4594-b9c1-ac608eda842f-000000%2FpL5U2UJcog124gv7q_7Yx6as76Dd-gkFQfaGIRtx4jw%3D224&data=05|02|alex_paine@baylor.edu|5537a85f84b441c587ca08dddef4fc97|22d2fb35256a459bbcf4dc23d42dc0a4|0|0|638911865991287290|Unknown|TWFpbGZsb3d8eyJFbXB0eU1hcGkiOnRydWUsIlYiOiIwLjAuMDAwMCIsIlAiOiJXaW4zMiIsIkFOIjoiTWFpbCIsIldUIjoyfQ%3D%3D|0|||&sdata=w0uyrKbxCdU1dcFYT3ajU7Mh9f5UEnKGkqiCYvjNdCw%3D&reserved=0).

We look forward to receiving your revised manuscript.

Kind regards,

Mary Diane Clark, PhD
Academic Editor
PLOS ONE

Journal Requirements:

If the reviewer comments include a recommendation to cite specific previously published works, please review and evaluate these publications to determine whether they are relevant and should be cited. There is no requirement to cite these works unless the editor has indicated otherwise. 

Please review your reference list to ensure that it is complete and correct. If you have cited papers that have been retracted, please include the rationale for doing so in the manuscript text, or remove these references and replace them with relevant current references. Any changes to the reference list should be mentioned in the rebuttal letter that accompanies your revised manuscript. If you need to cite a retracted article, indicate the article’s retracted status in the References list and also include a citation and full reference for the retraction notice.

Additional Editor Comments:

Thank you for the major revision. It makes the manuscript clearer. I am not going to send it out for a second review but I do have a few things that I need you to address prior to recommending publication.

**Point 1:** In general—you have extremely long sentences. –give it a look a see if some of them can become two or more sentences. One example is below.

Very end of page 3 to 4
Comprising roughly 10% of tenure-eligible faculty across the UC system, the promotion criteria for TP/PoT faculty reflect that of the Research Professor (which we define as the traditional academic tenure-track faculty position that is evaluated primarily on the strength of their research program) but with a greater emphasis placed on the value of teaching excellence (Harlow, et. al., 2020, UC 63 University of California, 2020).

Above is a long sentence—if you could take out the definition of traditional academic tenure track faculty it would be easier to parse

**Response:** This specific sentence has been shortened into two separate sentences, and the definition of Research professor has been removed. Additionally, the manuscript has been thoroughly read to locate and address other long sentences, and these have been adjusted for ease of reading.

**Point 2:** Page 4 line 71
Influenced by---add the by
Page 5 83-84
In particular, research on TP/PoT faculty indicates that they are currently doing so 84 within the UC system
Please be more specific about currently doing

**Response:** This appears to be a comment on the original manuscript rather than the revised. This edit was made within the revised manuscript and remains revised in this resubmission (theoretical framework paragraph 1). Additionally, the manuscript has been thoroughly read through to check for any additional grammatical errors/missing text such as this.

**Point 3:** Page 5 97-98
it is key that we better understand the role the perspective of TP/PoTs 98 themselves.
Not sure what you mean by the role the perspective

**Response:** Thank you for pointing out the lack of clarity within this sentence. As the remainder of the paragraph details this, we have removed this unnecessary and unclear sentence from the manuscript.

**Point 4:** Page 14—why not include the t value with the significance level
What p level did you use given the multiple t tests?
You need to correct the alpha for multiple tests
Same comment for Table 3—did you correct for increased t tests?

**Response:** Thank you for this comment. We revised Table 2 to include *t*-values and degrees of freedom alongside the p-values. We used α = .05 and applied a Holm correction for the six planned comparisons. After correction, expected scholarship (*p* = .006) and actual service (*p* < .001) remained significant, while actual scholarship (*p* = .044) did not.

We revised Table 3 to include *t*-values and degrees of freedom alongside the p-values. We used an α level of .05 and applied a Holm correction for the nine planned comparisons. After correction, the differences in DBER (*p* < .001) and K–12 professional development (*p* = .005) remained significant, while the smaller effects did not.

**Point 5:** I still don’t see the need to take up journal space with the large non-significant effects in Tables 5 and 6
If seems that statements that specific variables are not significant would be enough
I strongly recommend using the tables you now have in the supplemental files witht eh ANOVAs for each and moving these models in to the supplemental data.

I think that readers of this paper will find that more informative

**Response:** Thank you for this helpful suggestion. We have moved the full regression models (previously Tables 5 and 6) to the supplemental materials (now S2 and S4 Tables). In the main text, we summarize only the significant predictors and note that all others were non-significant, while highlighting the Type III ANOVA results to confirm the unique contributions.

[Note: HTML markup is below. Please do not edit.]

[NOTE: If reviewer comments were submitted as an attachment file, they will be attached to this email and accessible via the submission site. Please log into your account, locate the manuscript record, and check for the action link "View Attachments". If this link does not appear, there are no attachment files.]

While revising your submission, please upload your figure files to the Preflight Analysis and Conversion Engine (PACE) digital diagnostic tool, [https://pacev2.apexcovantage.com/](https://nam02.safelinks.protection.outlook.com/?url=https%3A%2F%2Ftrack.editorialmanager.com%2FCL0%2Fhttps%3A%252F%252Fpacev2.apexcovantage.com%252F%2F1%2F010f0198c14e5a17-81576c75-4302-4594-b9c1-ac608eda842f-000000%2FvFsBek4HV666w_lOCDXHvK9uIJV__bvWtYfwa-AqkcI%3D224&data=05|02|alex_paine@baylor.edu|5537a85f84b441c587ca08dddef4fc97|22d2fb35256a459bbcf4dc23d42dc0a4|0|0|638911865991302531|Unknown|TWFpbGZsb3d8eyJFbXB0eU1hcGkiOnRydWUsIlYiOiIwLjAuMDAwMCIsIlAiOiJXaW4zMiIsIkFOIjoiTWFpbCIsIldUIjoyfQ%3D%3D|0|||&sdata=q8LNtWkC2vaM1u24nLt1m9x3jV7MwcT1dBVjTmQhu9w%3D&reserved=0). PACE helps ensure that figures meet PLOS requirements. To use PACE, you must first register as a user. Registration is free. Then, login and navigate to the UPLOAD tab, where you will find detailed instructions on how to use the tool. If you encounter any issues or have any questions when using PACE, please email PLOS at [figures@plos.org](mailto:figures@plos.org). Please note that Supporting Information files do not need this step.
